# Anaphylactic Shock Following Administration of Iodinated Computed Tomography (CT) Contrast Media: A Case Series

**DOI:** 10.7759/cureus.113223

**Published:** 2026-07-23

**Authors:** Raghad Asim Abdulla Ahmed Shilla, Nissar Shaikh, Umme Nashrah, Umm E Amara

**Affiliations:** 1 Medical Education, University of Medical Sciences and Technology, Khartoum, SDN; 2 Surgical Intensive Care, Hamad General Hospital, Doha, QAT; 3 Medicine, Deccan College of Medical Sciences, Hyderabad, IND; 4 Obstetrics and Gynecology, Deccan College of Medical Sciences, Hyderabad, IND

**Keywords:** anaphylactic shock, contrast-enhanced ct, hypersensitivity reaction, iodinated contrast media, iohexol

## Abstract

Anaphylactic shock is a severe and life-threatening allergic reaction that can result from contrast agents used in imaging procedures. Iodinated contrast media (ICM) are indispensable in diagnostic computed tomography (CT), yet they carry a small but serious risk of anaphylactic reactions. Even with low-osmolar, nonionic agents such as iohexol, severe hypersensitivity can occur unpredictably, including in patients with no prior history of allergic reactions. We report a retrospective case series of three patients who experienced anaphylactic shock following administration of ICM during CT imaging at Hamad Medical Corporation, Doha, Qatar. Cases were identified through clinical incident reporting and radiology department records. Anaphylaxis was diagnosed according to clinical criteria, requiring an acute onset of symptoms involving compromise of two or more organ systems such as cardiovascular, respiratory, or cutaneous, following intravenous (IV) contrast media exposure. Patient data were extracted from electronic medical records, including clinical notes, nursing observations, laboratory investigations, and imaging reports. Informed consent was obtained from patients, and the report was compiled in accordance with institutional ethical guidelines. Cases 1 and 3 involved female patients with breast cancer receiving iohexol for oncological staging. Case 2 included a male patient with multiple comorbidities who received iodixanol for cardiac evaluation. In all three cases, anaphylaxis developed shortly after IV contrast administration and was successfully managed with epinephrine, corticosteroids, antihistamines, and IV fluid administration. All patients achieved full recovery without long-term complications. These cases underscore the unpredictable nature of contrast-induced anaphylaxis and the critical importance of preprocedural risk assessment, emergency preparedness, and rapid administration of treatment. Even patients without identifiable risk factors may develop life-threatening reactions, emphasizing that all contrast-administering settings must maintain robust emergency protocols.

## Introduction

Anaphylactic shock is a severe, life-threatening allergic reaction that can result from exposure to contrast agents used in imaging procedures [1]. Reactions to iodine-based contrast media are a serious but relatively uncommon complication in radiology [2]. Such instances require urgent attention because of their tendency to progress to a life-threatening condition. Iodinated contrast media (ICM) are among the most widely used pharmacological agents in diagnostic radiology, administered an estimated 75 million times annually worldwide to enhance the visualization of soft tissues, vascular structures, and tumors on computed tomography (CT) imaging. Despite their widespread and generally safe use, anaphylactic deaths still occur at a rate of approximately one to three per 100,000 to 1,000,000 administrations [1]. In breast cancer patients, CT imaging plays a vital role because it not only assesses the primary tumor but also evaluates the extent of metastatic disease, helps in monitoring the treatment response, and guides the treatment options [3]. Nonionic and low-osmolar contrast agents such as Omnipaque and Visipaque have a decreased risk of inducing severe reactions compared with high-osmolar agents that were used commonly in the past. Nevertheless, hypersensitivity reactions still remain an important clinical concern, ranging from mild urticaria to life-threatening anaphylactic shock. Therefore, proper management strategies should be understood to increase patient safety in radiology and oncology departments [2].

Anaphylaxis is an acute, hypersensitivity reaction that affects multiple body systems and is triggered by exposure to allergens, including medications, foods, and contrast agents. The pathophysiology of iodine-based contrast-induced anaphylaxis is very complex because it involves both immunologic (IgE-mediated) and nonimmunologic pathways (direct activation of mast cells and basophils) [4]. Once activated, these cells release inflammatory mediators like histamine, tryptase, and cytokines, which cause symptoms such as hypotension, bronchospasm, urticaria, and, in severe cases, shock [1]. Severe reactions occur in approximately 0.01%-0.04% of cases, and minor reactions in 3%-5% [1,4-6]. Although these rates are low, minor reactions can be unpredictable and may progress to severe outcomes. This is why it is advisable to exercise high caution with established emergency treatment algorithms in these treatment areas.

A particularly vulnerable group is cancer patients, who undergo frequent contrast-enhanced imaging for staging, treatment monitoring, and surveillance. Immune compromise secondary to malignancy itself, chemotherapy, and immunomodulatory therapies may alter susceptibility to hypersensitivity reactions [4]. Furthermore, repeated ICM exposure in such a population increases sensitization risk [2,4].

Recent studies highlight the need for rapid identification and management of anaphylactic reactions due to the potentially fatal outcomes of ICM-induced anaphylaxis. A consensus guideline by the American College of Radiology (ACR) (2023) recommends that intramuscular epinephrine should be used immediately as the first-line treatment. Contrast administration should be discontinued immediately, while simultaneous supportive measures, including oxygen supplementation and intravenous fluid resuscitation, should be initiated. Additional therapies, such as antihistamines and corticosteroids, may be administered as adjunctive treatment but should never delay epinephrine administration [7]. Radiology departments should be equipped with structured emergency response protocols for ICM-induced anaphylaxis, and staff should be appropriately trained to handle these situations. This, in turn, can improve outcomes in severe contrast-related reactions [8,9].

Recent research has increasingly focused on premedication strategies using corticosteroids and antihistamines in patients with prior hypersensitivity reactions to ICM. Although these regimens reduce the risk of recurrent hypersensitivity reactions, they do not provide complete protection, and severe breakthrough reactions, including anaphylaxis, may still occur [10].

Although severe reactions to ICM are rare, they continue to occur in routine clinical practice. We present three cases of anaphylactic shock following ICM administration during CT imaging, including two patients with breast cancer and one patient with multiple comorbidities. None had a known prior history of contrast-related reactions or food allergies; however, Case 2 had a documented penicillin allergy. Despite established management guidelines, detailed case series describing severe iodinated contrast-induced anaphylactic shock, particularly comparing different clinical presentations and management pathways, remain limited. These cases collectively illustrate the unpredictable nature of contrast hypersensitivity, the importance of swift emergency management, and the need for robust institutional treatment protocols, which are pivotal and fundamental to improving patient outcomes.

## Case presentation

This retrospective case series included all patients who developed severe iodinated contrast-induced anaphylactic shock following contrast-enhanced CT examinations at our institution between January 2024 and December 2025.

Case 1: breast cancer patient with no prior allergy history

An 88-year-old woman presented with a two-month history of a left breast lump. Clinical examination revealed notable skin tethering with mild nipple retraction. Imaging for further investigation was performed: bilateral mammogram and breast ultrasound (September 2024) demonstrated two highly suspicious left breast lesions. Breast MRI (September 2024) confirmed multiple suspicious masses and multiple left axillary lymph nodes.

Ultrasound-guided core biopsy (September 2024) confirmed invasive ductal carcinoma (no special type), with axillary nodal macrometastasis. A staging contrast-enhanced CT of the thorax, abdomen, and pelvis was subsequently scheduled.

Preprocedural assessment was unremarkable. The patient reported no history of allergies to food or medication, no prior contrast exposure, and no other comorbidities. Baseline creatinine was found to be 68 µmol/L (reference range: 62-106 µmol/L). Vital signs prior to the procedure were within normal limits (heart rate (HR), 68 bpm; blood pressure (BP), 121/74 mmHg; peripheral oxygen saturation (SpO₂), 96% on room air). A 22G intravenous (IV) cannula was placed in the right dorsal metacarpal vein. The CT scan commenced at 09:28 with IV administration of 100 mL iohexol (Omnipaque 300 mg/mL) at 2.5 mL/s.

Although the exact interval between contrast administration and symptom onset was not documented, within minutes of contrast injection, the patient developed nausea, vomiting, and coughing. Oxygen saturation dropped to 86%, and HR increased to 130 bpm. The contrast injection was stopped, and the rapid response team (RRT) was activated at 09:45. The patient was placed in the left lateral position, and oxygen was administered via a non-rebreather mask. On RRT arrival (09:48), vital signs were HR 124 bpm, BP 157/101 mmHg, and SpO₂ 100% on high-flow oxygen. IV hydrocortisone 200 mg and diphenhydramine 50 mg were administered immediately.

The patient was subsequently transferred to the recovery room. BP rapidly became unrecordable with only faint central pulses; the level of consciousness deteriorated. Epinephrine 0.5 mg was given intramuscularly at 09:55, and IV fluid resuscitation was initiated.

By 10:00, BP had improved to 91/57 mmHg with SpO₂ 100%; the patient began to respond. A further dose of epinephrine 0.1 mg IV was administered at 10:10 prior to transfer to the intensive care unit (ICU) at 10:19. ICU admission was undertaken because of profound hypotension requiring repeated epinephrine administration and close hemodynamic monitoring following cardiovascular collapse.

In the ICU, the patient was alert and spontaneously breathing on room air. Neurological status was intact (Glasgow Coma Scale (GCS), 15/15). Laboratory investigations demonstrated reactive leukocytosis, with the white blood cell count increasing from 5.7 x 10^3^/ µL before CT scan to 26.6 × 10³/µL (reference range: 4.0-11.0 × 10³/µL) and mild anemia of hemoglobin 9.9 g/dL (reference range: 12.0-15.5 g/dL), consistent with the acute event and underlying malignancy; an elevated serum tryptase of 82.7 mcg/L (reference range: <11.4 mcg/L), supporting the diagnosis of anaphylaxis. Venous blood gas analysis demonstrated a mildly alkalotic pH of 7.46 (reference range: 7.32-7.43) with an elevated lactate of 4.6 mmol/L (reference range: 0.5-2.2 mmol/L). Renal and hepatic parameters were normal.

Over the subsequent 24 hours, hemodynamics gradually normalized (Table 1). The patient was maintained on corticosteroids, antihistamines, IV fluids, and electrolyte replacement. She was discharged after 24 hours with a short course of prednisolone and antihistamines and was referred to the immunology clinic for further evaluation.

**Table 1:** Vital signs for Case 1 during ICU observation HR: heart rate; SBP: systolic blood pressure; DBP: diastolic blood pressure; SpO₂: oxygen saturation; RR: respiratory rate; RRT: rapid response team; NRBM: non-rebreather mask; ICU: intensive care unit

Time	HR (bpm)	SBP (mmHg)	DBP (mmHg)	SpO₂ (%)	O₂ therapy	RR (/minute)
08:54	68	121	74	96	None	Not recorded
09:45 (RRT activated)	130	110	71	86	NRBM	Not recorded
09:48 (RRT arrival)	124	157	101	100	NRBM	25
10:00	149	91	57	100	NRBM	Not recorded
13:00	72	92	61	98	Room air	17
15:00	68	101	57	98	Room air	17
20:00	84	106	64	97	Room air	18
07:00 (+24 hours)	76	118	65	100	Room air	21

Case 2: cardiovascular patient with penicillin allergy

The second patient was a 59-year-old man with a background of hypertension, chronic obstructive pulmonary disease (COPD), and ischemic heart disease (IHD), with a documented penicillin allergy characterized by generalized pruritus and gastrointestinal symptoms. He had no prior history of hypersensitivity reaction to contrast media. He was admitted in November 2025, for contrast CT-guided cardiac evaluation. Preprocedural assessment was unremarkable, with baseline vitals demonstrating a BP of 136/83 and a saturation of 96% on room air. Iodixanol (Visipaque 320 mg/mL) was selected as the contrast agent. IV access was established through the right antecubital vein using a 22G cannula, and 90 mL of contrast was administered at 2.5 mL/s.

During contrast administration, a reaction was noted, where the patient developed an erythematous rash with generalized skin flushing and swelling of the lips and oral mucosa. He reported dizziness and tingling in his fingers and was unable to maintain a semiupright position. Oxygen saturation decreased to 94% on room air, and BP fell to 105/58 mmHg. Although the exact interval between symptom onset and treatment was not documented, the contrast injection was stopped, and IV hydrocortisone 200 mg and diphenhydramine 50 mg were administered without delay.

The patient's condition deteriorated with progressive hoarseness of voice, worsening dyspnea, and even further hemodynamic compromise; BP declined to 71/38 mmHg. In response, epinephrine 0.5 mg was administered intramuscularly into the vastus lateralis. The patient was transferred to the emergency department for further stabilization. At the time of transfer, the airway was patent, and the patient was phonating without stridor; lip edema had resolved. Supplemental oxygen was administered, IV fluid resuscitation was initiated, and the patient was stabilized with continued supportive management. He did not require ICU admission (Table 2). Following stabilization in the emergency department, the patient remained hemodynamically stable with cutaneous symptoms over the subsequent hours. Laboratory investigations demonstrated reactive leukocytosis, with the white blood cell count increasing from 9.0 to 22.0 × 10³/µL (reference range: 4.0-11.0 × 10³/µL). Venous blood gas analysis showed a normal pH of 7.40 (reference range: 7.32-7.43) and a normal lactate of 1.4 mmol/L (reference range: 0.5-2.2 mmol/L). Serum tryptase was not obtained or documented during the acute event. He was discharged with a short course of oral corticosteroids and antihistamines, advised to avoid ICM in future imaging unless deemed clinically essential, and referral to allergy/immunology was recommended.

**Table 2:** Key clinical events and hemodynamic parameters for Case 2 HR: heart rate; SBP: systolic blood pressure; DBP: diastolic blood pressure; SpO₂: oxygen saturation; IV: intravenous; IM: intramuscular; NRBM: non-rebreather mask; RA: room air

Time	HR (bpm)	SBP (mmHg)	DBP (mmHg)	SpO₂ (%)	Intervention
Baseline	74	136	83	96% RA	IV access established
During contrast injection	90	105	58	94%	Hydrocortisone 200 mg IV + Diphenhydramine 50 mg + NRBM
Postinjection (deterioration)	99	71	38	Declining	Epinephrine 0.5 mg IM; emergency transfer + NRBM

Case 3: breast cancer patient with confirmed elevated serum tryptase

The third patient was a 47-year-old woman with an identical oncological background to Case 1: left breast invasive ductal carcinoma confirmed on core biopsy with axillary nodal macrometastasis. Staging contrast-enhanced CT of the thorax, abdomen, and pelvis was requested (March 20, 2025).

Preprocedure screening showed no previous history of contrast reactions, no known drug or food allergies, and no active comorbidities. The patient had not previously received ICM for any imaging study. Baseline hemodynamics and renal function were normal. The CT scan commenced with IV administration of 100 mL iohexol (Omnipaque 300 mg/mL) at 2.5 mL/s.

Shortly after the commencement of contrast injection, the patient became acutely unwell with nausea and vomiting. Although the exact interval between symptom onset and treatment was not documented, the contrast injection was stopped. The RRT was activated at 09:45 and arrived at 09:49. At that time, vital signs were as follows: HR 125 bpm, BP 80/50 mmHg, SpO₂ 86% on nasal cannula, and RR 25/minute. Chest auscultation demonstrated bilateral equal air entry without wheeze. IV hydrocortisone 100 mg and diphenhydramine 50 mg were administered. No cutaneous manifestations were observed; gastrointestinal symptoms predominated throughout the reaction.

The patient was moved from the CT scanner to the recovery room. Rapid deterioration occurred instantly, with the BP becoming unrecordable; the carotid pulse was present, but the radial pulse was absent; and the patient became unarousable. Epinephrine 0.5 mg was administered intramuscularly, and IV Ringer's lactate was commenced as a bolus. Neurological and hemodynamic improvement was evident within five minutes, with BP recovering to 90 mmHg systolic and SpO₂ returning to 100% on 15 L/minute oxygen. The patient began opening her eyes and making sounds but remained agitated.

A further dose of IV epinephrine (0.1 mg) was administered because of persistent hemodynamic instability and the severity of the anaphylactic reaction. Given the need for repeated epinephrine administration, the patient was transferred to the ICU for close hemodynamic monitoring and observation. On arrival to the ICU, hemodynamics and GCS steadily improved without the need for further vasopressors. Serum tryptase, measured in the acute phase, was elevated at 40.30 mcg/L (reference range: <11.4 mcg/L), providing biochemical confirmation of mast cell activation consistent with anaphylaxis. Venous blood gas analysis demonstrated a normal pH of 7.38 (reference range: 7.32-7.43) and a lactate of 1.7 mmol/L(reference range: 0.5-2.2 mmol/L). These findings indicated preserved acid-base status without significant tissue hypoperfusion despite the patient's profound hemodynamic instability. Prior to discharge, the patient was advised to avoid future iodinated contrast exposure unless clinically essential, and a formal allergy/immunology referral was made. The clinical events and parameters for this case are summarized in Table 3.

**Table 3:** Key clinical events and hemodynamic parameters for Case 3 HR: heart rate; SBP: systolic blood pressure; DBP: diastolic blood pressure; SpO₂: oxygen saturation; RRT: rapid response team; NRBM: non-rebreather mask; IM: intramuscular; IV: intravenous; ICU: intensive care unit

Time	HR (bpm)	SBP (mmHg)	DBP (mmHg)	SpO₂ (%)	Intervention
Baseline	79	131	74	99%	-
09:45 (RRT activated)	125	110	69	85%	Lateral position, O₂
09:49 (RRT arrival)	125	80	50	86%	Hydrocortisone 100 mg + Diphenhydramine 50 mg IV+ Nasal cannula for O_2_
Post RRT	140	Unrecordable	Unrecordable	100% 15L O₂	Epinephrine 0.5 mg IM; Ringer's stat + NRBM
<5 minute postadrenaline	Not recorded	90	Not recorded	100%	NRBM
Pretransfer	149	96	54	100%	Epinephrine 0.1 mg IV+ NRBM
ICU arrival	109	110	55	100%	No further adrenaline required

Results

**Figure 1:**
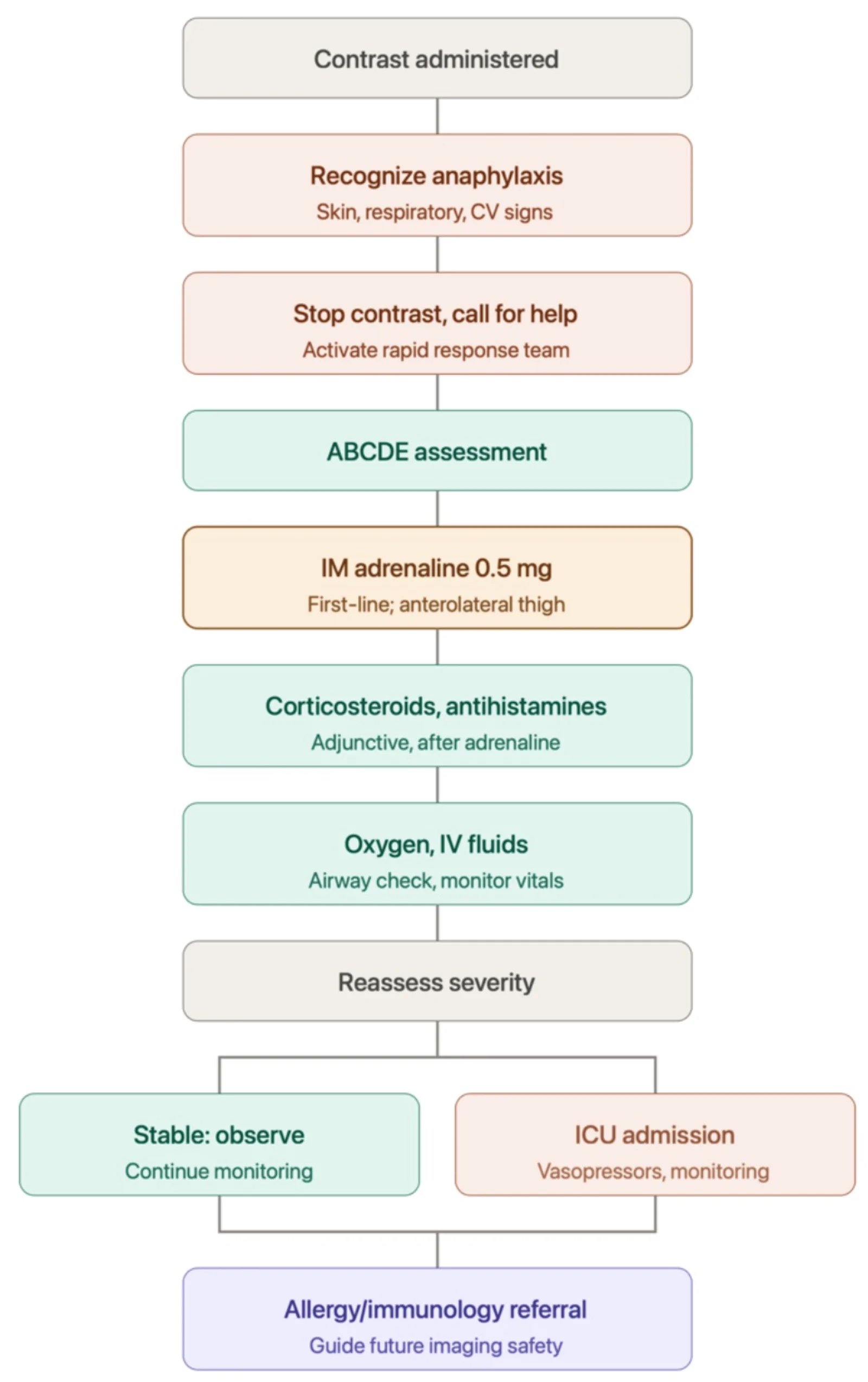
Clinical management algorithm for suspected iodinated contrast-induced anaphylaxis CV: cardiovascular; IM: intramuscular; IV: intravenous; ICU: intensive care unit; ABCDE: Airway, Breathing, Circulation, Disability, and Exposure

All three patients developed anaphylactic shock following contrast administration, with no prior history of contrast hypersensitivity. Onset was rapid in all cases, occurring during or immediately after the injection. The clinical spectrum ranged from cutaneous and hemodynamic compromise (Case 2) to cardiovascular collapse with loss of consciousness (Cases 1 and 3). Epinephrine was required in all three cases, and ICU admission was necessary in two. All patients survived and were discharged without permanent sequelae. Table 4 provides a comparative overview, and the corresponding clinical management algorithm for suspected iodinated contrast-induced anaphylaxis is summarized in Figure 1.

**Table 4:** Comparative summary of the three cases IDC: invasive ductal carcinoma; HTN: hypertension; COPD: chronic obstructive pulmonary disease; IHD: ischemic heart disease; BP: blood pressure; SpO₂: oxygen saturation; LOC: level of consciousness; IM: intramuscular; IV: intravenous; ICU: intensive care unit; ED: emergency department

Feature	Case 1	Case 2	Case 3
Age/sex	88/female	59/male	47/female
Underlying condition	Breast cancer (IDC)	HTN, COPD, IHD	Breast cancer (IDC)
Known contrast allergy	No	No	No
Known drug or food allergy	None	Yes, drug (penicillin)	None
Contrast agent	Iohexol (Omnipaque 300)	Iodixanol (Visipaque 320)	Iohexol (Omnipaque 300)
Volume/rate	100 mL/2.5 mL/s	90 mL/2.5 mL/s	100 mL/2.5 mL/s
Presenting features	Vomiting, ↓SpO₂, ↓LOC, hypotension	Rash, angioedema, dizziness, ↓SpO₂, hoarseness, hypotension	Vomiting, ↓LOC, hypotension
Lowest recorded BP	Unrecordable	71/38 mmHg	Unrecordable
Lowest SpO₂	86%	94%	85%
Contrast stopped	Yes	Yes	Yes
Oxygen and IV fluids	Yes	Yes	Yes
IM epinephrine first line and route	0.5 mg IM; 0.1 mg IV	0.5 mg IM	0.5 mg IM; 0.1 mg IV
ICU admission	Yes	No (emergency transfer)	Yes
Tryptase level	82.7 mcg/L (elevated) (reference: <11.4)	Not documented	40.30 mcg/L (elevated) (reference: <11.4)
Outcome	Full recovery; ICU stabilization	Full recovery; stabilized in ED, no ICU admission required	Full recovery; ICU stabilization
Allergy referral	Yes	Recommended	Yes

## Discussion

This case series collectively illustrates several key clinical aspects of contrast-induced anaphylaxis, including early recognition, prompt management, and institutional preparedness, which merit careful consideration by radiologists, oncologists, and emergency clinicians.

During the two-year study period, approximately 72,000 contrast-enhanced CT examinations were performed at our institution. Only three patients developed severe iodinated contrast-induced anaphylactic shock, corresponding to an institutional incidence of approximately 0.0042% (4.2 cases per 100,000 examinations, or roughly one case per 24,000 contrast-enhanced CT examinations). This figure is consistent with previously reported rates of severe immediate hypersensitivity reactions to nonionic ICM, cited in the literature at 0.01%-0.04% of administrations [1,4,5].

Despite this rarity, the unpredictability of anaphylaxis in the setting of negative screening is the most noticeable feature. All three cases had no prior history of contrast allergy. Standard preprocedural screening, including allergy history and renal function assessment, was performed in each case and yielded no contraindications. Beyond confirming these reactions are rare, our series also demonstrates that these uncommon reactions can present with markedly different clinical manifestations despite exposure to modern low-osmolar contrast agents. Two patients progressed rapidly to cardiovascular collapse requiring ICU admission, whereas one patient was stabilized without intensive care despite significant hypotension. This variability reinforces that the severity of anaphylaxis cannot be predicted solely by the type of contrast agent, previous allergy history, or initial clinical presentation, emphasizing the need for immediate recognition and preparedness in all patients receiving ICM [1,5].

Existing risk factors, such as prior ICM reactions, asthma, multiple allergies, or previous anaphylaxis, are associated with a higher relative risk for reaction recurrence, but the majority of severe reactions occur in patients without these factors. Case 2 uniquely presented a patient with a penicillin allergy and significant cardiorespiratory comorbidity (COPD, IHD), which are risk factors for worse clinical outcomes, rather than for the reaction being triggered in the first place [7]. Nonetheless, similar severe reactions were observed in Cases 1 and 3 without any such background. Paradoxically, Case 2, the only patient with significant cardiorespiratory comorbidity, had the least severe clinical course, requiring only a single dose of intramuscular epinephrine and never progressing to cardiac arrest or ICU-level care, whereas Cases 1 and 3, both without cardiovascular disease, progressed to unrecordable BP and loss of consciousness. This observation should be interpreted cautiously given the small sample size, but it illustrates that theoretical outcome-risk factors do not reliably predict comparative severity.

Furthermore, agent-specific risk data from Kim et al. [1] identified iopromide as carrying significantly higher odds of hypotension/shock (OR 3.09) compared with any other agents. None of our three patients received iopromide (two received iohexol and one iodixanol), yet all three developed severe hypotensive/hypoxic reactions. This suggests that agent-specific risk profiles derived from single-center cohorts may not generalize reliably, and that no currently available contrast agent can be considered free of anaphylaxis risk.

The pathophysiology of ICM-induced anaphylaxis is not fully characterized. Both immunologic (IgE-mediated) and nonimmunologic pathways contribute. Nonionic low-osmolar agents such as iohexol are associated with a lower incidence of adverse reactions than older high-osmolar agents, because they primarily cause less complement activation and direct mast cell degranulation [1,4]. Nevertheless, as demonstrated in all three cases, severe reactions can still occur. In Cases 1 and 3, the elevated serum tryptase of 82.7 and 40.30 mcg/L, respectively, provided important biochemical evidence of mast cell degranulation and supports a diagnosis of true anaphylaxis rather than a vasovagal or anxiety-mediated event. Measurement of serum tryptase within one to three hours of symptom onset is recommended by international guidelines for the laboratory confirmation of anaphylaxis; its availability in Cases 1 and 3 adds diagnostic value that was absent in Case 2 and should be sought whenever feasible following suspected anaphylaxis.

In the acute setting, several alternative diagnoses were considered, including vasovagal reactions, anxiety-related events, acute coronary syndromes, pulmonary embolism, and contrast extravasation. However, the rapid onset immediately following iodinated contrast administration, multisystem involvement, profound hypotension, and prompt clinical improvement following treatment with intramuscular epinephrine strongly supported the diagnosis of anaphylaxis according to established diagnostic criteria. In Cases 1 and 3, markedly elevated serum tryptase further provided biochemical confirmation of mast cell activation, supporting the clinical diagnosis of anaphylaxis and making alternative diagnoses such as vasovagal syncope or anxiety-related reactions less likely.

Overall, management in our cases was largely consistent with current ACR recommendations, particularly with respect to rapid discontinuation of contrast administration, oxygen supplementation, IV fluid resuscitation, and administration of intramuscular epinephrine [7]. However, our cases also highlight an important learning point. In Cases 1 and 3, corticosteroids and antihistamines were administered before epinephrine during the initial response. Although epinephrine was subsequently administered promptly following hemodynamic deterioration, current guidelines recommend intramuscular epinephrine as the first-line treatment and advise against delaying its administration in favor of adjunctive medications [7,8]. Initial administration of IV hydrocortisone and diphenhydramine alone, before epinephrine, in the earlier phase of Cases 1 and 3 highlights a common pitfall: corticosteroids have a delayed onset of action and should never replace or precede epinephrine in anaphylaxis management. Departments must ensure that all staff are trained to recognize when corticosteroids alone are insufficient and to escalate to epinephrine without any delays [8,9]. These cases, therefore, illustrate a common real-world challenge and reinforce the importance of regular staff education and protocol adherence.

Structured RRT response contributed to the rapid stabilization in Cases 1 and 3, with activation within minutes of symptom onset and arrival within three to four minutes. While RRT activation and arrival times were rapid in Cases 1 and 3 (within three to four minutes), response speed alone is an incomplete measure of system effectiveness. In both cases, the patient progressed to cardiovascular collapse after RRT arrival, coinciding with an initial treatment sequence that prioritized corticosteroids and antihistamines over epinephrine. Furthermore, Case 2 demonstrates that severe reactions may also occur in settings where ICU-level care is not immediately adjacent, reinforcing the requirement that all contrast-administering environments, not only radiology departments, maintain emergency medications and trained personnel capable of managing anaphylaxis to the point of transfer.

Clinical severity varied considerably between cases. Patients in Cases 1 and 3 required ICU admission because of cardiovascular collapse, repeated epinephrine administration, and the need for close hemodynamic monitoring, whereas Case 2 stabilized after a single dose of intramuscular epinephrine and supportive treatment without requiring intensive care. This highlights that escalation of care should be guided by the patient's hemodynamic status rather than the presence of cutaneous manifestations alone. Following stabilization, all three patients were discharged on a short course of oral corticosteroids and antihistamines. None developed a delayed or biphasic reaction during the observation period. Patients in Cases 1 and 3 were referred for allergy/immunology follow-up, whereas for Case 2, referral to an allergy/immunology specialist was not documented, representing a deviation from current recommendations. All patients with suspected iodinated contrast-induced anaphylaxis should undergo specialist evaluation to guide future imaging and reduce the risk of recurrent reactions.

For patients requiring future contrast-enhanced imaging, management should involve a multidisciplinary team including radiologists, oncologists, and immunologists. Premedication may reduce the risk or severity of recurrent reactions but does not eliminate the possibility of breakthrough anaphylaxis. When appropriate, alternative imaging modalities should be considered, while patients who require iodinated contrast may benefit from evaluation for alternative contrast agents or desensitization in specialized centers.

These three cases, occurring in the same institution over a relatively short period, highlight the importance of system-level preparedness. Emergency anaphylaxis kits containing epinephrine (both intramuscular and IV formulations), corticosteroids, antihistamines, and IV fluids should be immediately accessible in all areas where contrast is administered. Staff competency in anaphylaxis recognition and management should be formally assessed and refreshed regularly. Incident reporting and case review, as undertaken here, are essential quality improvement mechanisms that facilitate learning and protocol refinement.

Limitations

This study has several limitations. It is a retrospective case series from a single institution, and only three patients developed contrast-induced anaphylaxis during the study period. Consequently, the findings may not be generalizable to other institutions or populations. Laboratory confirmation with serum tryptase was available in only two of the patients; long-term allergy follow-up was incomplete for one patient. Nevertheless, detailed chronological documentation and comprehensive clinical management provide valuable educational insights into the recognition and treatment of severe iodinated contrast-induced anaphylaxis.

## Conclusions

This case series of iohexol- and iodixanol-induced anaphylactic shock illustrates that contrast hypersensitivity can develop unexpectedly in patients without a history of allergic reactions or any other recognized risk factors. Early identification of symptoms and rapid administration of intramuscular epinephrine remain key to improving morbidity and mortality in such situations. In accordance with current guideline recommendations, all facilities administering ICM should have regular training for their staff for recognition of symptoms of anaphylaxis and have immediate access to resuscitation medications for emergency use. For patients undergoing frequent imaging procedures involving contrast agents, weighing the benefits of diagnosis against the risks of an adverse reaction is necessary. Premedication with corticosteroids and antihistamines may reduce the severity or likelihood of recurrent reactions, but it does not eliminate the risk of anaphylaxis, and breakthrough reactions can still occur despite premedication. When feasible, acute and baseline serum tryptase measurements should be obtained after potential anaphylactic reactions to support the diagnosis biochemically and allow further immunological investigations.
